# Recurrent nocturnal hypoglycaemia as a cause of morning fatigue in treated Addison’s disease – favourable response to dietary management: a case report

**DOI:** 10.1186/s12902-015-0058-6

**Published:** 2015-10-24

**Authors:** Kristina S Petersen, R. Louise Rushworth, Peter M Clifton, David J Torpy

**Affiliations:** Dietitian, School of Pharmacy and Medical Sciences, University of South Australia, GPO Box 2471, Adelaide, SA 5000 Australia; School of Medicine, Sydney, The University of Notre Dame, 60 Oxford St., Darlinghurst, NSW 2010 Australia; School of Pharmacy and Medical Sciences, University of South Australia, GPO Box 2471, Adelaide, SA 5000 Australia; Endocrine and Metabolic Unit, Royal Adelaide Hospital, University of Adelaide, North Terrace, Adelaide, SA 5000 Australia

**Keywords:** Addison’s disease, Hypoglycaemia, Fatigue, Dietary modification

## Abstract

**Background:**

Addison’s disease, or primary adrenal insufficiency, is often associated with reduced well-being and fatigue despite use of currently recommended adrenal hormone replacement. Hypoglycaemia is a known manifestation of glucocorticoid deficiency, but is generally considered rare in adults and not relevant to troubling ongoing symptoms in patients with Addison’s disease.

**Case presentation:**

A 43 year old woman with a three year history of Addison’s disease complained of severe morning fatigue and headaches, despite standard glucocorticoid replacement therapy in the form of thrice daily hydrocortisone and mineralocorticoid replacement with fludrocortisone. Alternative glucocorticoid replacement regimens and the addition of dehydroepiandrosterone replacement therapy had no effect. Nocturnal hypoglycaemia was suspected and a 4-day continuous glucose monitor system (CGMS) revealed hypoglycaemia (interstitial glucose < 2.2 mmol/L) between 0200–0400 h on 3 of 4 days. The patient was counselled to take an evening snack designed to ensure slow absorption of ingested carbohydrates. Nocturnal hypoglycaemia was then absent on follow up CGMS assessment. The patient noted a marked symptomatic improvement in morning symptoms, but with persistent fatigue during the day.

**Conclusion:**

Currently, the best strategy for control of non-specific symptoms in treated Addison’s disease is unknown, but it may be that investigation for hypoglycaemia and treatment, where necessary, could assist some sufferers to achieve improved wellbeing. A systematic study of this phenomenon in Addison’s disease is required.

## Background

Addison’s disease or primary adrenal insufficiency (PAI) is managed with adrenal hormone replacement (glucocorticoid and mineralocorticoid). Ongoing fatigue despite best practice hormone replacement is a clinical problem [1, 2]. Treated patients are not regarded as being at risk of hypoglycaemia despite loss of the glucose elevating effects of glucocorticoids (GC) [3]. We describe a patient with nocturnal hypoglycaemia diagnosed on continuous glucose monitoring that had resolved with a late evening dietary supplement with attendant improvement in morning fatigue and headache. This problem may be more frequent than recognised and a systematic study is required.

### Case presentation

The patient was a 43 year old Caucasian woman who had a three year history of diagnosed Addison’s disease (PAI). At the time of presentation with PAI, she reported weight loss of at least 4 kg over 6 months, fatigue and hyperpigmentation. Biochemistry revealed a serum sodium of 132 mmol/L (R 137–145 mmol/L) and potassium of 5.4 mmol/L (R 3.5–4.9 mmol/L), plasma cortisol of 16 nmol/L and an ACTH level of 1097 pmol/L (R 10–60 pmol/L). A 250mcg ACTH stimulation test showed a non-significant rise in serum cortisol from baseline 16 nmol/L to 17 nmol/L (R >500 nmol/L) at both 30 and 60 min. The plasma renin concentration was > 500 uIU/mL (R 7–50 uIU/mL) and plasma aldosterone <30 pmol/L (110–860 pmol/L). Primary hypothyroidism was evident with a TSH of 46 mIU/mL (R 0.5-4.0 mIU/mL) and a free T4 of 10 nmol/L (R 10–25 nmol/L). Elevated thyroid peroxidase antibodies and a firm small goitre were consistent with Hashimoto’s disease. Twelve months before diagnosis of PAI, mild normocytic anaemia was detected; Hb 89 g/L and biochemically confirmed iron deficiency (ferritin 12 μg/L [15–250 μg/L], transferrin saturation 6 % [10-35 %], transferrin 2.79 g/L [2.0-4.0 g/L], with normal B12 and folate levels. Oral iron supplements led to recovery of anaemia and normal iron studies. At the time of diagnosis of PAI she was found to have strongly positive endomysial and gliadin antibodies, consistent with coeliac disease, confirmed on jejunal biopsy. Adrenal hormone replacement included hydrocortisone (HC), initially in a thrice daily regimen at 0700 h, 1200 h, 1600 h in doses of 10/10/4 mg respectively, fludrocortisone 0.1 mg daily (after initial lower doses due to oedema), thyroxine 75mcg daily and a gluten free diet. Follow up revealed improved well-being, normalization of electrolytes, haemoglobin, plasma renin and TSH, as well as resolution of coeliac antibodies and a normal jejunal biopsy.

However, ongoing fatigue prompted trials of various GC regimens. Prednisolone 5 mg in the morning and 1–2 mg afternoon (0700 h, 1400 h) was not effective in relieving fatigue and a thrice daily regimen (0600 h, 1145 h, 1700 h) of HC 10/8/5 mg, equating to a total dose of 0.44 mg/kg or 15 mg/m^2^ was employed, encompassing the period where CGMS measures were later performed. A trial of dehydroepiandrosterone (DHEA) was used in an attempt to alleviate the patient’s fatigue in doses up to 25 mg daily with normalization of DHEA levels that were initially undetectable. Unfortunately, this resulted in subtle hirsutism with hair growth around the chin and sideburn area, and the dose was reduced by the patient down to 5 mg daily; overall there was no consistent effect of DHEA on wellbeing.

No definite cause for the patient’s ongoing fatigue could be determined on clinical grounds. A particular characteristic of the fatigue was its severity and timing, occurring each morning and described by the patient as “extreme” “very hard to get up” “like being hit by a train” subsequently taking approximately 20 min to significantly resolve, after the usual morning HC and breakfast. There were also frequent non-migrainous morning headaches. There was no consistent excessive nocturnal wakening but poor or broken sleep was encountered on occasion. There were no episodes of nocturnal diaphoresis.

**Fig. 1:**
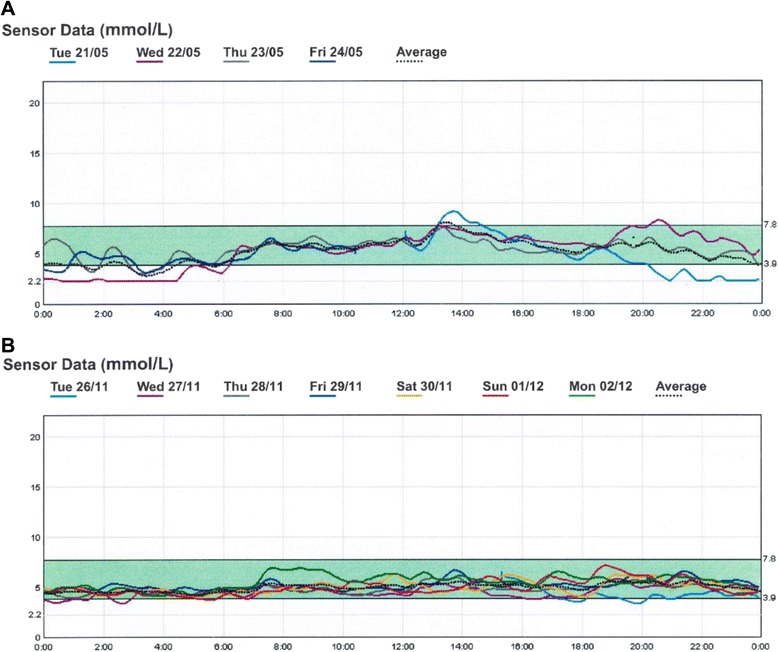
Continuous glucose monitoring before (**a**) and after (**b**) the dietary manipulation in the patient with treated Addison’s disease

Hypoglycaemia was suspected and a continuous glucose monitor system (CGMS) was used to collect interstitial glucose levels over four days (Fig 1a). The CGMS revealed hypoglycaemia on three of four days tested, which was lasted up to six hours throughout one night. Calibration was performed against finger prick blood glucose levels (BGL), which were performed four times daily (i.e. before each meal and before bed). Dietary manipulation to address the hypoglycaemia was attempted and the patient was advised to have an evening low glycaemic index (GI) snack incorporating a fat load; specifically a whole-grain rice cake or multi-grain gluten free bread topped with cheese, cream cheese or peanut butter. The patient consumed this snack between 2030–2100 h during a repeat period of CGMS assessment, which was performed over seven days. The repeat CGMS measurements (Fig. 1b) showed resolution of the hypoglycaemia. The CGMS values were validated as per usual practice against finger prick capillary blood glucose values. The dietary changes resulted in resolution of headaches and improved energy after waking, although there was persistent daytime fatigue.

## Discussion

This patient had a background of PAI, concomitant primary hypothyroidism due to Hashimoto’s disease, and coeliac disease, consistent with autoimmune polyglandular syndrome type 2 (APS-2). Treatment included GC and mineralocorticoid replacement, thyroxine and a gluten free diet. Severe morning fatigue and headaches were noted and CGMS revealed recurrent prolonged nocturnal hypoglycaemia. The use of an evening dietary intervention alleviated symptoms and CGMS evidence of hypoglycaemia. We suggest that PAI patients with symptoms such as marked fatigue, headaches or unexplained diaphoresis, particularly if nocturnal or on waking, be screened for hypoglycaemia and, if present, dietary measures may provide a simple strategy for improved well-being.

Physical and mental fatigue, stress, anxiety and impaired concentration (“L’encephalothie addisonne”) are well recognized symptoms of adrenal insufficiency (AI) that are associated with many aspects of reduced quality of life in patients and often persist despite optimal treatment [1, 2]. It has been suspected that the use of oral GCs, which necessarily lead to cortisol levels that do not precisely mimic the normal circadian rhythm or produce brief ultradian pulses of cortisol secretion may underlie impaired quality of life in those with PAI.

Retrospective studies of individuals taking long acting GCs such as prednisolone and dexamethasone have suggested a predisposition towards reduced well-being in AI patients [4] in addition to an increased risk of lower bone mineral density [5], increased hip fracture rates [6] and higher levels of abdominal adiposity with dysplipidaemia [7]. These results have prompted a trend towards the use of short-acting, low dose HC rather than longer acting synthetic GCs [8, 9]. However, these low dose, short-acting HC schedules invoke both variable cortisol levels [10] and nocturnal hypocortisolism due to the duration of action of HC, plasma disappearance half-life 90 mins, duration of effect 3 h [11, 12]. Nocturnal hypocortisolism may be exacerbated by the use of short-acting HC, which combined with the effects of fasting, may increase the risk of hypoglycaemia. Episodic hypocortisolism has the potential to increase the risk of adrenal crises as there is an epidemiological association in one study between increased use of short-acting GC and increased rates of adrenal crises, but prospective studies of this association are not available and other factors may have contributed to apparent rising rates of adrenal crises in recent years [8, 9].

Late dosing of GCs is sometimes associated with sleep difficulties, presumably due to the known effects of GCs on arousal [13]. Hence, studies of GC regimens have focussed on short-acting GCs given at various frequencies, but avoiding administration 4–6 h before bedtime, in an attempt to mimic circadian plasma cortisol levels and improve short-term non-blinded measures of quality of life. Two studies have shown that circadian cortisol rhythms are closer to physiological if HC is given on a thrice-daily 10/5/5 mg schedule [14, 15]. Weight related dosing reduces inter-individual variation in cortisol levels [16]. Cortisone acetate, which is converted to HC endogenously, has been shown to produce greater ACTH suppression and more physiological cortisol levels in Addison’s when given as a thrice rather than twice daily regimen [17, 18]. A four-dose regimen of HC produced more physiological circadian cortisol levels than 2-dose, but quality of life was similar [19, 20]. A practical limitation is the patients reported difficulty in adherence to frequent dose schedules [21]. Overall, although these studies have shown that more frequent GC dosing can more closely mimic physiological cortisol levels there is limited short term data on quality of life and the optimum GC replacement regimen in PAI is not known.

Studies of two types of sustained release HC, once daily dual release Plenadren and twice daily enteric coated Chronocort, have shown more physiological circadian cortisol profiles than regular HC [22]. In the case of Plenadren, metabolic studies have revealed lower HbA1c, waist circumference and LDL cholesterol despite similar cortisol peaks and integrated values and lower ACTH levels in patients with Addison’s switching from HC to Plenadren [23]. Blinded controlled studies, using a visually identical rapid release HC preparation to assess the effect on well-being, have not been performed [24, 25]. Improved quality of life has been reported in patients receiving HC via an unblinded subcutaneous infusion on a circadian schedule, although quality of life did not change in one placebo-controlled double-blind study [26, 27].

Administration of DHEA, an adrenal steroid that is not secreted in appreciable amounts in PAI, has been shown to have variable effects on wellbeing and quality of life in several studies, but the results of a meta-analysis did not demonstrate a consistent benefit and for this reason DHEA is not recommended routinely [28].

Hypoglycaemia is a known manifestation of adrenal insufficiency (AI), particularly among children [29] but is generally considered rare in adults and not relevant to troubling ongoing symptoms such as fatigue and reduced vitality in treated AI. Patients with concomitant type 1 diabetes and PAI are commonly reported to have reduced insulin requirement and some guidelines have suggested that longer acting GCs such as prednisolone be used in this setting [30].

Acute cortisol withdrawal in PAI patients leads to reduced hepatic glucose output and increased glucose oxidation [3]. Quantitatively, the effect of hypococortisolism on glycaemia is large; experimentally induced hypocortisolism in PAI patients was met with a requirement to increase glucose infusion rates by 70 % to maintain a predetermined blood glucose [3]. Relatively small doses of hydrocortisone in the evening may alleviate hypoglycaemia, since the glucose elevating effect of hydrocortisone is more marked after evening than morning dosing of hydrocortisone [31]; the mechanism is also time related with an immediate reduction in insulin secretion followed in 4–12 h by increased insulin resistance [32, 33].

One study has examined the question of the prevalence of hypoglycaemia in PAI, using CGMS technology [34]. Of 13 patients screened one patient, a 46 year old man with PAI and no other comorbidities, had nocturnal hypoglycaemia of approximately one hour duration reaching a trough of 2.6 mmol/L. The hypoglycaemia resolved on repeat CGMS assessment with administration of the last HC dose in the late evening. In this case the apparent symptoms were frequent waking at night, which settled with the delayed HC administration.

## Conclusions

We suggest that the use of CGMS assessment in selected cases may help detect hypoglycaemia and that simple and safe dietary manipulations similar to that described above, may help to resolve the hypoglycaemia. Alternative strategies may include the administration of GC later in the day, such as the evening, however there is a risk of disturbed sleep as discussed above. We suggest that further studies are required, perhaps focussing on those with early morning symptomatology, to determine the frequency of hypoglycaemia in patients with PAI. When detected, appropriate dietary manipulation or the use of long-acting glucococorticoids may allow improvement in symptoms, an important issue as reduced quality of life and wellbeing occur in a substantial proportion of patients with PAI.

## Consent

Written informed consent was obtained from the patient for publication of this Case report and any accompanying images. A copy of the written consent is available for review by the Editor of this journal.

## Abbreviations

ACTH: Adrenocorticotropic hormone
AI: Adrenal insufficiency
APS-2: Autoimmune polyglandular syndrome type 2
BGL: Blood glucose levels
CGMS: Continuous glucose monitor system
DHEA: Dehydroepiandrosterone
GC: Glucocorticoid
GI: Glycaemic index
HC: Hydrocortisone
PAI: Primary adrenal insufficiency
TSH: Thyroid-stimulating hormone
QID: Four times per day

## References

[1] Løvås K, Loge JH, Husebye ES (2002). Subjective health status in Norwegian patients with Addison’s disease. Clin Endocrinol.

[2] Meyer G, Hackemann A, Penna-Martinez M, Badenhoop K (2013). What affects the quality of life in autoimmune Addison's disease?. Horm Metab Res.

[3] Christiansen JJ, Djurhuus CB, Gravholt CH, Iversen P, Christiansen JS, Schmitz O, Weeke J, Jørgensen JOL, Møller N (2007). Effects of cortisol on carbohydrate, lipid, and protein metabolism: studies of acute cortisol withdrawal in adrenocortical failure. J Clin Endocrinol Metab.

[4] Bleicken B, Hahner S, Loeffler M, Ventz M, Decker O, Allolio B, Quinkler M (2010). Influence of hydrocortisone dosage scheme on health‐related quality of life in patients with adrenal insufficiency. Clin Endocrinol.

[5] Løvås K, Gjesdal CG, Christensen M, Wolff AB, Almås B, Svartberg J, Fougner KJ, Syversen U, Bollerslev J, Falch JA (2009). Glucocorticoid replacement therapy and pharmacogenetics in Addison's disease: effects on bone. Eur J Endocrinol.

[6] Björnsdottir S, Sääf M, Bensing S, Kämpe O, Michaelsson K, Ludvigsson JF (2011). Risk of hip fracture in Addison’s disease: a population‐based cohort study. J Intern Med.

[7] Filipsson H, Monson JP, Koltowska-Häggström M, Mattsson A, Johannsson G (2006). The impact of glucocorticoid replacement regimens on metabolic outcome and comorbidity in hypopituitary patients. J Clin Endocrinol Metab.

[8] Rushworth RL, Torpy DJ (2015). Modern hydrocortisone replacement regimens in adrenal insufficiency patients and the risk of adrenal crisis. Horm Metab Res.

[9] Rushworth RL, Torpy DJ (2015). Adrenal insufficiency in Australia: is it possible that the Use of lower dose, short-acting glucocorticoids has increased the risk of adrenal crises?. Horm Metab Res.

[10] Simon N, Castinetti F, Ouliac F, Lesavre N, Brue T, Oliver C (2010). Pharmacokinetic evidence for suboptimal treatment of adrenal insufficiency with currently available hydrocortisone tablets. Clin Pharmacokinet.

[11] Czock D, Keller F, Rasche FM, Häussler U (2005). Pharmacokinetics and pharmacodynamics of systemically administered glucocorticoids. Clin Pharmacokinet.

[12] Meikle AW, Tyler FH (1977). Potency and duration of action of glucocorticoids: effects of hydrocortisone, prednisone and dexamethasone on human pituitary-adrenal function. Am J Med.

[13] Fietta P (2006). Glucocorticoids and brain functions. Rivista Di Biologia.

[14] Peacey SR, Guo CY, Robinson AM, Price A, Giles MA, Eastell R, Weetman AP (1997). Glucocorticoid replacement therapy: are patients over treated and does it matter?. Clin Endocrinol.

[15] Howlett TA (1997). An assessment of optimal hydrocortisone replacement therapy. Clin Endocrinol.

[16] Mah PM, Jenkins RC, Rostami‐Hodjegan A, Newell‐Price J, Doane A, Ibbotson V, Tucker GT, Ross RJ (2004). Weight‐related dosing, timing and monitoring hydrocortisone replacement therapy in patients with adrenal insufficiency. Clin Endocrinol.

[17] Laureti S, Falorni A, Santeusanio F (2003). Improvement of treatment of primary adrenal insufficiency by administration of cortisone acetate in three daily doses. J Endocrinol Investig.

[18] Barbetta L, Dall’Asta C, Re T, Libe R, Costa E, Ambrosi B (2005). Comparison of different regimens of glucocorticoid replacement therapy in patients with hypoadrenalism. J Endocrinol Investig.

[19] Ekman B, Bachrach‐Lindström M, Lindström T, Wahlberg J, Blomgren J, Arnqvist HJ (2012). A randomized, double‐blind, crossover study comparing two‐and four‐dose hydrocortisone regimen with regard to quality of life, cortisol and ACTH profiles in patients with primary adrenal insufficiency. Clin Endocrinol.

[20] Alonso N, Granada ML, Lucas A, Salinas I, Reverter J, Oriol A, Sanmartí A (2004). Evaluation of two replacement regimens in primary adrenal insufficiency patients. Effect on clinical symptoms, health-related quality of life and biochemical parameters. J Endocrinol Investig.

[21] Forss M, Batcheller G, Skrtic S, Johannsson G (2012). Current practice of glucocorticoid replacement therapy and patient-perceived health outcomes in adrenal insufficiency-a worldwide patient survey. BMC Endocr Disord.

[22] Debono M, Ghobadi C, Rostami-Hodjegan A, Huatan H, Campbell MJ, Newell-Price J, Darzy K, Merke DP, Arlt W, Ross RJ (2009). Modified-release hydrocortisone to provide circadian cortisol profiles. J Clin Endocrinol Metab.

[23] Giordano R, Guaraldi F, Marinazzo E, Fumarola F, Rampino A, Berardelli R, et al. Improvement of anthropometric and metabolic parameters, and quality of life following treatment with dual-release hydrocortisone in patients with Addison’s disease. *Endocrine* 2015:1–9. 10.1007/s12020-015-0681-z 26184416

[24] Mallappa A, Sinaii N, Kumar P, Whitaker MJ, Daley L-A, Digweed D, Eckland DJ, VanRyzin C, Nieman LK, Arlt W (2014). A phase 2 study of chronocort®, a modified-release formulation of hydrocortisone, in the treatment of adults with classic congenital adrenal hyperplasia. J Clin Endocrinol Metab.

[25] Johannsson G, Bergthorsdottir R, Nilsson AG, Lennernas H, Hedner T, Skrtic S (2009). Improving glucocorticoid replacement therapy using a novel modified-release hydrocortisone tablet: a pharmacokinetic study. Eur J Endocrinol.

[26] Øksnes M, Björnsdottir S, Isaksson M, Methlie P, Carlsen S, Nilsen RM, Broman J-E, Triebner K, Kämpe O, Hulting A-L (2014). Continuous subcutaneous hydrocortisone infusion versus oral hydrocortisone replacement for treatment of Addison's disease: a randomized clinical trial. J Clin Endocrinol Metab.

[27] Gagliardi L, Nenke MA, Thynne TR, von der Borch J, Rankin WA, Henley DE, Sorbello J, Inder WJ, Torpy DJ (2014). Continuous subcutaneous hydrocortisone infusion therapy in Addison’s disease: a randomized, placebo-controlled clinical trial. J Clin Endocrinol Metab.

[28] Alkatib AA, Cosma M, Elamin MB, Erickson D, Swiglo BA, Erwin PJ, Montori VM (2009). A systematic review and meta-analysis of randomized placebo-controlled trials of DHEA treatment effects on quality of life in women with adrenal insufficiency. J Clin Endocrinol Metab.

[29] Hsieh S, White PC (2011). Presentation of primary adrenal insufficiency in childhood. J Clin Endocrinol Metab.

[30] Elbelt U, Hahner S, Allolio B (2009). Altered insulin requirement in patients with type 1 diabetes and primary adrenal insufficiency receiving standard glucocorticoid replacement therapy. Eur J Endocrinol.

[31] Plat L, Leproult R, L’Hermite-Baleriaux M, Fery F, Mockel J, Polonsky KS, Van Cauter E (1999). Metabolic effects of short-term elevations of plasma cortisol Are more pronounced in the evening than in the morning 1. J Clin Endocrinol Metab.

[32] Van Cauter E, Leproult R, Kupfer DJ (1996). Effects of gender and age on the levels and circadian rhythmicity of plasma cortisol. J Clin Endocrinol Metab.

[33] Plat L, Byrne MM, Sturis J, Polonsky KS, Mockel J, Fery F, Van Cauter E (1996). Effects of morning cortisol elevation on insulin secretion and glucose regulation in humans. Am J Physiol.

[34] Meyer G, Hackemann A, Reusch J, Badenhoop K (2012). Nocturnal hypoglycemia identified by a continuous glucose monitoring system in patients with primary adrenal insufficiency (Addison’s disease). Diabetes Technol Ther.

